# From waste to nutrition: valorization strategies for surplus and ugly produce within the Food Use and Waste Hierarchy

**DOI:** 10.3389/fnut.2026.1868657

**Published:** 2026-07-22

**Authors:** Irene Domínguez, Marina Astudillo-Pascual, Roxana Tudor, José Luis del Río, Víctor Ortiz-Somovilla, Emma Cantos-Villar

**Affiliations:** 1 Andalusian Institute for Research and Training in Agriculture, Fisheries, Food and Ecological Production (IFAPA), IFAPA La Mojonera, Almería, Spain; 2 IFAPA Servicios Centrales, Sevilla, Spain; 3 IFAPA Alameda del Obispo, Córdoba, Spain; 4 IFAPA Rancho de la Merced, Cádiz, Spain

**Keywords:** bioactive compounds, circular economy, food loss, fruits, policies, prevention, vegetables

## Abstract

Fruit and vegetables (F&Vs) account for approximately 50% of global food loss and waste (FLW). This represents an environmental and economic burden, as well as a significant, yet often overlooked concern for human nutrition. At the farm level, surplus (products for which there is no buyer) and “ugly produce” (products rejected for failing aesthetic standards) are often discarded or diverted for low value uses, such as composting or animal feed, despite retaining a good nutritional and functional quality. Current valorization frameworks, including the EU Food Use and Waste Hierarchy (FUWH), prioritize environmental and economic criteria, yet largely overlook an explicit nutrition-sensitive dimension. This review addresses this gap by proposing a nutrition-sensitive reinterpretation of the EU FUWH that is specifically applied to surplus and ugly F&Vs at the primary production level. The key advance over previous adaptations of the hierarchy is the incorporation of a tier aimed at “food-grade processing and nutritional valorization” above animal feed use. This new tier is based on the principle that retaining nutrients and bioactive compounds for human consumption is a higher-order outcome. The situation reveals a nutrition-sensitive paradox in which strategies with the highest nutritional preservation potential face the greatest implementation barriers, calling for policy, economic and logistical interventions that realign incentive structures with nutrition-sensitive outcomes.

## Graphical Abstract

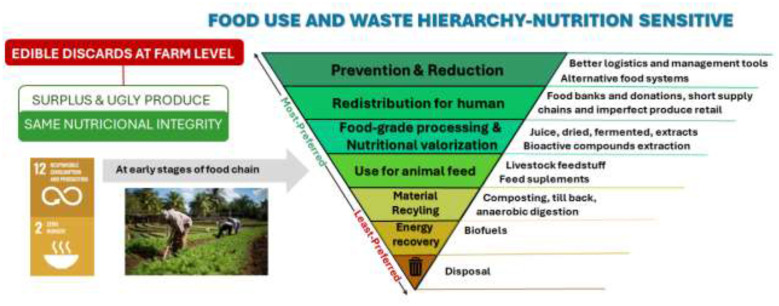

## 1 Introduction

Food loss and waste (FLW) accounts for spillage or leakage of food across the entire food supply chain (FSC). When addressing the FLW problem, it is imperative to address conceptualization and dimension issues. According to the Food and Agriculture Organization of the United Nations (FAO) (1), the boundary between food loss (FL) and food waste (FW) is conceptually placed at specific stages of the FSC where they occur. FL takes place from primary production to processing, and FW at retail and consumer stages. Though such distinction may sound straightforward, there are still some gaps in the international legislation regarding standardized definitions and quantification approaches. The 2030 Agenda for Sustainable Development of the United Nations (UN) and the Farm to Fork Strategy (F2F) of the European Commission (EC) put the focus on the imperative need of reducing FLW, however they do not get to the heart of the problem conceptualization.

Regarding the FLW dimension, global estimates published by the FAO (1) and the United Nations Environment Programme (UNEP) (2) suggest that 14% of global food production is lost between harvest and retail. A further 17% is wasted at retail, food service, and household levels. More recent data from the European Union (EU) confirm this trend. According to Eurostat (3), a total of approximately 58 million tons of food were wasted in the EU in 2023, equaling 130 kg per capita per year, with primary production and manufacturing accounting for 28% (36 kg per capita). Country-level data reveal a fourfold variation, ranging from 65 kg per capita in Spain to 286 kg in Cyprus, with the primary production ranging from 9% in Portugal to 55% in Denmark of total food waste, underscoring the need for country-specific implementation frameworks. The economic value of this waste is estimated at 132 billion euros (4). This represents a daunting scenario, especially when considering that approximately 30% of the global population suffers from moderate to severe food insecurity (5). Beyond its environmental and economic implications, FLW also represents a significant challenge for human nutrition, as large amounts of edible and nutrient-rich foods are removed from the FSC.

The nutritional consequences of F&V loss are not uniformly distributed across regions or population groups. In high-income countries, farm-level F&V losses represent primarily a missed economic and environmental opportunity; however, in regions with persistent food insecurity, including Sub-Saharan Africa, South Asia, and Central America, the loss of edible F&Vs at primary production level may reduce the micronutrient availability. Globally, an estimated one in two preschool-aged children and two in three women of reproductive age suffer from micronutrient deficiencies, predominantly in low- and middle-income countries (6).

A further obstacle is the lack of standardized quantification methods for primary production losses. Current FLW estimates are conservative and uncertain, as field level discard is systematically excluded from major datasets such as Eurostat (3), leading to a significant underestimation of the problem (7–9). The absence of reliable data on the volume and nutritional status of discarded F&Vs represents a major barrier to prioritizing valorization strategies and evaluating the dietary impact of recovery interventions.

According to Gillman et al. (10) the most abandoned edible food at the farm level falls into two broad categories. The first category is surplus, which encompasses perfect produce for which there may not be a buyer, typically due to overproduction or supply-demand imbalances (11). The second category, “ugly produce,” also referred to as suboptimal, imperfect, or cosmetically rejected produce, consists of edible food that fails to meet quality or aesthetic standards due to non-desirable size, shape, color and surface defects (12, 13).

Unfortunately, to mitigate the risk of buyer rejection, potential financial losses, and disposal costs, farmers rather abandon the imperfect produce beforehand in the field (14). With all this, F&Vs constitute approximately 50% of global FLW, making them a prime target for mitigation strategies (15). Focusing on the ugly produce category, F&Vs are particularly noteworthy. Porter et al. (16) estimated that over one-third of European farm production (51 million tons per year) is discarded due to aesthetic considerations. More recently, Eurostat data (3) confirm that primary production alone generated approximately 5.6 million tons of food waste across the EU in 2023. The largest absolute volumes came from France (~1.3 million tons), Poland (763,509 tons), Spain (756,517 tons), and Romania (613,337 tons), all major F&V-producing countries. However, as mentioned above, these numbers certainly underestimate the true scale of losses, since Eurostat's methodology excludes unharvested or pre-market rejected produce.

Traditionally, valorization strategies within the agri-food system have been developed to manage by-products and residues generated during food processing, which are often inedible and nutritionally depleted. In contrast, surplus or ugly produce may retain their full nutritional potential at the time of discard. Despite this, surplus and aesthetically imperfect F&Vs are frequently managed using the same frameworks and recovery pathways as inedible by-products. This lack of differentiation can result in the premature downgrading of edible foods to low-value applications, such as composting or disposal, thereby forfeiting opportunities to preserve dietary nutrients, bioactive compounds, and public health benefits.

In this context, the European Union Food Use and Waste Hierarchy (EU FUWH) offers a structured approach to prioritize food loss management options; however, its application in primary production has largely focused on environmental efficiency rather than nutritional outcomes (17, 18). Surplus and ugly produce are two categories explicitly distinguished from “non-edible” fractions (parts of the plant not consumed, such as roots, cores, or seeds), “non-recoverable” material (food that has deteriorated beyond safe consumption), and agri-food by-products or processing residues (peel, pomace, etc.), which should occupy different positions in valorization frameworks. Therefore, we propose a nutrition-oriented interpretation of the FUWH, in which strategies are evaluated not only according to their position within the hierarchy, but also based on their capacity to retain nutritional quality and contribute to human diets.

Previous reviews on food waste valorization (17–19) have predominantly focused on environmental, economic, and resource efficiency perspectives within circular economy and policy contexts, whereas explicitly nutrition-sensitive interpretations of food waste hierarchies, particularly in relation to the retention of nutritional and functional compounds, remain limited. To address this gap, this review proposes a nutrition-sensitive reinterpretation of the EU FUWH specifically applied to surplus and ugly produce, incorporating food-grade processing and bioactive compounds recovery as a distinct, high-priority valorization tier, a categorization absent from current official frameworks.

## 2 Nutritional and functional potential of surplus and ugly produce

Surplus and ugly produce may have comparable levels of vitamins, minerals, and bioactive compounds (20) and their exclusion from markets represents a missed opportunity not only to improve nutrient intake but also to reduce FL and promote more sustainable dietary patterns. A balanced and varied diet, tailored to individual needs and rich in F&Vs, is recommended (21). Excluding these products from supply chains reduces food availability and may also negatively impact on dietary quality, especially among populations with limited access to fresh F&Vs. Therefore, it is crucial to consider the nutritional and functional potential of the discarded products to support nutrition-sensitive valorization strategies and prevent nutrient loss in food systems.

F&Vs are major contributors to micronutrient intake, providing both essential vitamins and minerals. They are rich in vitamin C, folate, and provitamin A carotenoids, as well as important minerals such as potassium and magnesium. While vitamins from F&Vs play crucial roles in antioxidant defense and cellular function, their mineral content is particularly relevant for maintaining electrolyte balance, regulating blood pressure, and supporting metabolic processes (22).

Dietary fiber represents another valuable nutritional component of F&Vs. Both soluble and insoluble fiber fractions remain largely unaffected by visual defects. These fibers support gut health, glycemic regulation, and cardiovascular risk reduction (23, 24). Given the widespread inadequacy of dietary fiber intake (25), redirecting fiber-rich discarded produce toward human food applications is warranted.

Beyond their basic nutritional composition, discarded edible produce is a valuable source of functional compounds with established health-promoting properties. These include phenolics, carotenoids, and other antioxidants characteristic of plant-based products. Such compounds have been associated with a reduced risk of non-communicable diseases (26–28), multiple beneficial biological effects (29–31), and improved mental health outcomes (32, 33).

Phenolic compounds, including flavonoids, anthocyanins, phenolic acids, and tannins, are widely distributed in F&Vs and contribute to antioxidant, anti-inflammatory, and cardioprotective effects among others (34–37). Long-term consumption of diets rich in polyphenols offers protection against the development of various chronic diseases, including cardiovascular diseases, cancer, diabetes, and inflammatory disorders. Carotenoids such as β-carotene, lutein, and lycopene are key bioactive compounds associated with visual health, immune function, and reduced oxidative stress (38–40).

According to Lee and Kader (20), as confirmed later by other authors (29, 41–43), the primary determinants of the micronutrient, fiber, and phytochemical content of F&Vs are cultivar, maturity at harvest, and agronomic conditions. Notably, while cosmetic defects do not impair nutritional quality *per se*, the environmental stressors responsible for some imperfections may in fact stimulate the accumulation of protective secondary metabolites, potentially enhancing the phytochemical value of imperfect produce (41, 42). However, direct comparative studies on the nutritional composition of cosmetically rejected vs. market-grade produce remain scarce, representing an important gap in literature and a priority area for future research.

Overall, off-grade produce can be considered nutritionally equivalent to commercial-grade products at the point of discard. The primary risk to their nutritional quality arises not from their initial characteristics, but from delays in decision-making and inappropriate valorization pathways that accelerate postharvest degradation processes (43, 44).

In conclusion, the recovery of nutritional and functional compounds from discarded F&Vs before degradation represents a strategic opportunity to bridge circular economy approaches and nutrition-sensitive food policies. Valorization pathways that preserve these compounds can help safeguard their health-promoting potential while supporting food system decisions guided by both nutritional and socioeconomic criteria.

## 3 Nutrition-sensitive valorization strategies across the Food Use and Waste Hierarchy

The concept of waste prioritization has evolved progressively within the food systems' context. The EU Waste Framework Directive 2008/98/EC, as amended by Directive (EU) 2018/851, established the foundational waste hierarchy, comprising five tiers: prevention, preparing for re-use, recycling, other recovery (including energy recovery), and disposal, as a general system applicable across all waste streams (45, 46). Later, Papargyropoulou et al. (11) proposed one of the first food-specific adaptations, distinguishing between surplus and waste, as well as between avoidable and unavoidable waste, while emphasizing the prioritization of higher-value uses such as human consumption. Building on this approach, Teigiserova et al. (19), proposed a seven-tier hierarchy within the context of the circular bioeconomy, distinguishing material recycling, nutrient recovery, and energy recovery as separate levels.

**Figure 1:**
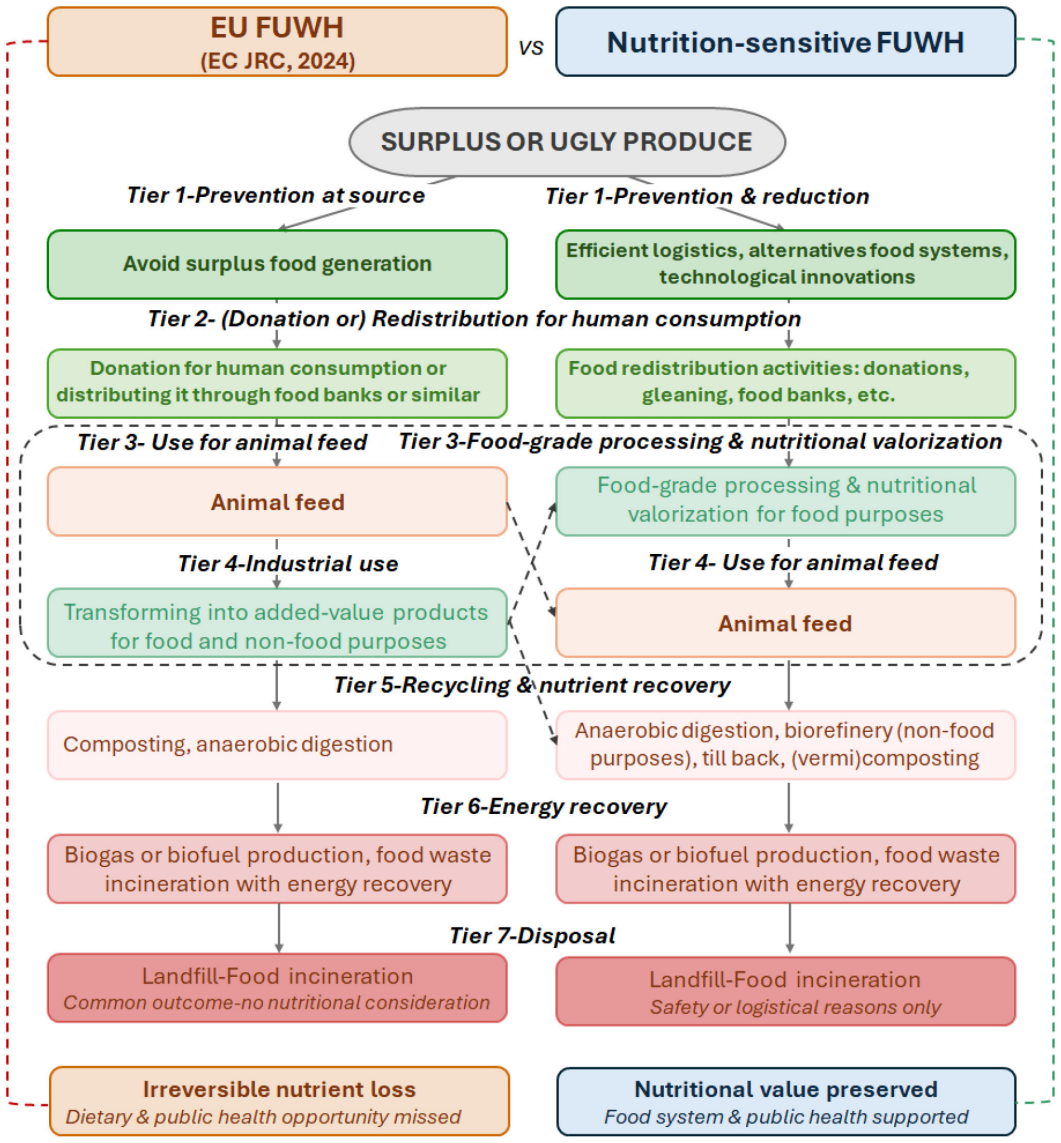
Comparison between the EU Food Use and Waste Hierarchy (EC JRC, 2024) and the proposed nutrition-sensitive Food Use and Waste Hierarchy.

In 2020, different authors on behalf of the European Commission's Joint Research Center (EC JRC), defined the Food Use Hierarchy (47), which was then updated and refined to distinguish between prevention and waste management actions, to the current EU FUWH (48) covering prevention at source, donation or redistribution for human consumption, use as animal feed, industrial uses, recycling and nutrient recovery, energy recovery, and disposal, as illustrated in Figure 1.

Within this framework, edible food is expected to be directed toward higher-tier options, particularly redistribution, while inedible fractions are diverted to lower-tier valorization pathways. However, in practice, and particularly at primary production level, surplus and ugly produce that retains full nutritional value is disproportionately left unharvested, composted, or diverted to animal feed, rather than being directed toward human consumption channels (7, 49). This misalignment between hierarchical priorities and actual practices may result in avoidable losses of edible nutritional value from the human food supply.

To our knowledge, the preceding frameworks explicitly did not establish the transformation of edible discarded produce as a distinct valorization category. In the EU FUWH, processing approaches such as thermal preservation (canning, pasteurization), freezing, drying, or transformation into minimally processed, ready-to-eat, or dehydrated and powdered format have no explicit placement. The redistribution tier primarily addresses direct food redistribution for human consumption and does not explicitly establish a separate category for food-grade processing of edible discarded produce, while the industrial uses tier is defined by non-food technical applications which reflect the environmental orientation of the hierarchy. This is coherent within the hierarchy's original waste-management scope but is less suitable when the objective is to evaluate edible discarded produce through a nutrition-sensitive lens.

As illustrated in Figure 1, the nutrition-sensitive interpretation proposed in this review refines the EU FUWH by redefining and repositioning the valorization tier with an explicit nutritional lens. The conventional “industrial uses” tier, which encompassed applications ranging from biofuels and biochemicals to other non-food industrial uses, but without explicit nutritional criteria for human consumption, is here reconsidered and repositioned above animal feed as “food-grade processing and nutritional valorization.”

The rationale for this repositioning is based on the recognition that edible discarded produce retains nutritional value, and that directing it toward human consumption, even after transformation, preserves more of its direct human dietary value than diverting it to animal feed or non-food industrial applications. This consideration is not explicitly captured into current FUWH decision-making tools.

The following sections describe each tier of the nutrition-sensitive FUWH and the valorization strategies available at the primary production level.

### 3.1 Prevention and reduction

Prevention and reduction, at the apex of the pyramid, are widely understood as the implementation of measures to reduce FLW at its source. From a nutrition-sensitive perspective, the primary objective of this tier is to maintain the edibility and nutritional integrity of food, preventing its premature downgrading within the food system (50). Efficient logistics and management are pivotal to addressing FL in the early stages of the FSC, particularly in primary production.

Enhancing pre- and postharvest practices, including production and management, transportation, storage, handling, processing and packaging procedures, represents an early and effective approach to reducing produce losses (51–53) and to preserve the nutritional and phytochemical quality of fresh produce. Indeed, these health-promoting compounds are highly labile, and their content can decline sharply when produce is exposed to inadequate temperature management, mechanical damage, or prolonged transit times (54). Phytochemicals are particularly sensitive to such processes as polyphenol oxidase activity, chlorophyllase mediated chlorophyll breakdown, and light or heat-induced carotenoid degradation can all proceed rapidly under suboptimal postharvest conditions (55). Studies tracking African indigenous leafy vegetables along supply chains in Kenya demonstrated that chlorophyll and carotenoid concentrations fell by 71–91% and 71–92%, respectively, between harvest and market, while dry matter, minerals, and protein were reduced by 3–29%, depending on the county and handling conditions (56).

In this context, shortening supply chains emerges as a particularly effective strategy to reduce both quantitative and nutritional losses. In this sense, alternative food systems (AFSs) have been widely discussed within this prevention framework to reduce FL in primary production by shortening supply chains and strengthening direct producer–consumer connections (19, 57, 58). By facilitating earlier redistribution and greater flexibility in quality standards, these models can help retain nutritionally intact F&Vs within the human food chain. Models such as short food supply chains (SFSCs), community supported agriculture (CSA), farmers' markets, and direct sales initiatives can enhance resource efficiency, improve coordination, and facilitate the commercialization and acceptance of imperfect produce (53, 59, 60).

In parallel, some growers have developed collaborative arrangements to share information and collectively invest in infrastructure such as cooling and storage facilities, supporting more effective surplus management (61). From a nutritional standpoint, shared storage and cooling infrastructure can delay quality deterioration and nutrient losses, enabling timely valorization decisions. More complex and still limited social innovations, including local hybrid food hubs (HFHs), further contribute to these approaches by providing centralized logistical and operational services that enable efficient and sustainable localized supply chains (53).

In addition, technological innovations can further strengthen prevention strategies. For instance, imaging-based tools can support the early identification of ugly produce at both warehouse and field levels (62), as well as improve crop yield prediction (63, 64), thereby facilitating proactive surplus management before nutritional quality is compromised.

Investing in fundamental components such as training, infrastructure, and tailored logistical solutions constitutes a critical initial step toward reducing FL in primary production. Beyond these measures, the primary sector could significantly benefit from the adoption of social innovations and technological advancements. However, the implementation of the latter faces several challenges, including substantial initial cost. In many cases, these measurements require upfront investment that small and medium size enterprises (SMEs) may struggle to accommodate or are just difficult to acquire.

Overall, prevention and direct human consumption not only minimize FL but also maximize nutritional benefits, positioning this tier at the top of a nutrition-sensitive interpretation of the FUWH. Strategies that maintain surplus and aesthetically imperfect produce within the human food chain preserve their full nutritional profile and support increased F&V intake, which remains below recommended levels in many populations.

### 3.2 Redistribution for human consumption

Tier 2, as in previous hierarchy versions, encompasses redistribution for human consumption (48), which pertains to the redirection of unharvested or unsold edible produce toward human consumption with the dual objective of reducing FL and promoting food security (65, 66). It is a broad category that includes several food redistribution activities (FRAs) such as donations, gleaning, and food banks, among others (67).

Gleaning occurs when F&Vs left in the field after harvest are collected by volunteers or organized groups and redirected for human consumption. Gleaning activities are part of non-profit organizations, networks composed of volunteers, or social enterprise projects, such as the case of the “Espigoladors” in Catalonia (Spain). This activity, when properly organized, can minimize the need for farmers to invest additional time, effort, or personnel. On the other hand, food donations represent an already known and consolidated strategy to mitigate FL in several EU countries, such as Italy, whose government actively work to facilitate and promote food donation practices (68).

Despite the benefits of these activities and the incentives, a significant proportion of farmers remain unwilling to participate in FRAs, mainly because of health standards and product liability, bureaucracy and profitability, supply/demand imbalances, and logistical issues, among others.

The supply-demand imbalance presents a distinct challenge. Donations of agricultural producers often do not align with the fluctuating needs of food banks. These discrepancies result in periods of either oversupply or deficiencies. This issue is aggravated by logistical limitations, including insufficient storage and transport capacity, and the difficulty of coordinating FRAs' voluntary labor, as highlighted by Akkerman et al. (69). Although these challenges are particularly evident in food donation systems, they may also affect other FRAs, including gleaning initiatives. In this matter, Facchini et al. (70) proposed addressing these issues through the creation of a large distribution system, supported and sustained through national governments and stakeholders. While this top-down approach is promising, it may require significant time to become operational.

When conducted properly, gleaning may offer practical advantages for some farmers than donations, as it can reduce on-farm workload and limit the need for additional labor or time commitment. As part of the reuse for human consumption, FRAs play a crucial dual role in addressing FL and advancing social objectives by promoting food security and community welfare. However, it seems that their full potential can only be realized through targeted interventions, including additional efforts to simplify administrative processes and offer more transparent and effective financial incentives.

### 3.3 Food-grade processing and nutritional valorization

When direct consumption is not feasible due to logistical, temporal, or market constraints, food-grade processing and nutritional valorization offer a secondary yet nutritionally relevant valorization pathway. Unlike non-food applications, these strategies retain surplus and ugly produce within the human nutrition domain, either as processed foods or as sources of functional ingredients.

**Figure 2:**
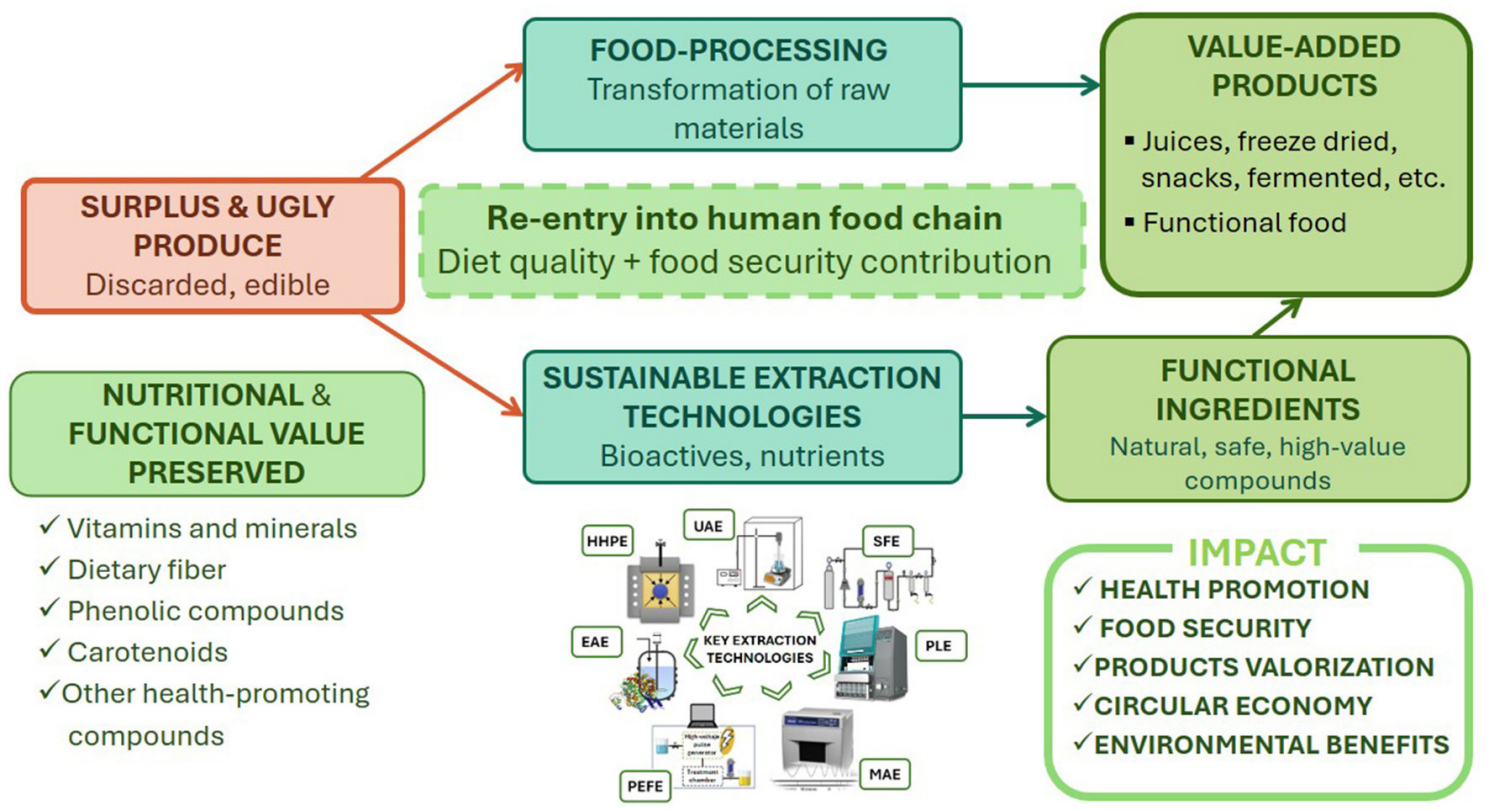
Proposed valorization framework for surplus and ugly produce through food-grade processing and sustainable extraction of bioactive and nutrients, enabling the production of high-value products that can be reintroduced into the human food chain, thus supporting improved diet quality and food security.

Unlike its conventional classification within the material recycling tier of the EU FUWH, food-grade processing and nutritional valorization is repositioned here as a high-priority strategy whenever extracted bioactive compounds are directed toward human nutrition (Figure 2).

Food-grade transformation of surplus and ugly produce encompasses a wide range of processing formats, from freezing and thermal sterilization to minimally processed and dehydrated products, each with distinct implications of technology requirements, elaboration procedures and nutritional retention. Vitamin C, highly sensitive to heat, oxygen and mechanical damage, can serve as reliable indicator of overall nutrient preservation across processing methods (71). In this sense, freezing represents the most effective conventional method for retaining vitamin C, with losses occurring primarily during storage rather than at the processing stage itself. Thermal stabilization and canning results in losses ca. of 50% while minimally processed fresh present significant losses at processing stage (up to 25%) driven by mechanical damage and enzyme activation. On the other hand, non-thermal technologies applicable to ready-to-eat processing offer superior vitamin C retention compared to conventional heat treatment. High pressure processing (HPP) achieves retention of 67–99% for different F&Vs, consistently outperforming thermal pasteurization. Pulse electric fields (PEF) show high but more variable retention, ranging from 85–98% in orange-juice and gazpacho to as low as 40% in some matrices depending on process parameters, highlighting the importance of process optimization for each product (72).

This tier also contemplates the extraction of functional components, including phenolic compounds, carotenoids, dietary fiber fractions, and natural pigments, enabling the development of value-added ingredients for functional food development. These applications allow nutritionally rich discarded produce to contribute indirectly to human diets through fortified or reformulated foods (73).

For compound extraction, the conventional extraction techniques include Soxhlet extraction, maceration, hydrodistillation and cold pressing (74). However, these techniques are usually time-consuming and require large amounts of toxic solvents and energy (75). These challenges require the implementation of innovative, environmentally friendly extraction technologies to significantly improve process efficiency, by enhancing yield and extracting quality while reducing environmental impact (76–79).

Table 1 summarizes the main emerging extraction approaches proposed for phenolics and carotenoids extraction, along with representative yield and recovery data across different matrices. These techniques include ultrasound assisted extraction (UAE), supercritical fluid extraction (SFE), microwave-assisted extraction (MAE), pressurized liquid extraction (PLE), pulsed electric field extraction (PEFE), enzyme-assisted extraction (EAE), and high hydrostatic pressure extraction (HHPE).

**Table 1:** Summary of methods used for the extraction of bioactive compounds from F&Vs pomace.

Matrix	Target bioactive compounds	Recovery/Content (optimized)	References
Ultrasound-Assisted Exraction (UAE)			
Apple pomace	Quercetin, chlorogenic acid, gallic acid, phloretin, phloridzin, rutin	Quercetin: 1,628 μg/g; chlorogenic acid: 1,181 μg/g; gallic acid: 972 μg/g; TPC: 5.6 mg GAE/g	Rashid et al. (83)
Tomato pomace	Chlorogenic, caffeic, gallic, ferulic, p-coumaric acids, quercetin, catechin	Chlorogenic acid: 52.33 ± 0.80 μg/g Quercetin: 1.47 ± 0.11 μg/g Gallic acid: 14.4 ± 0.80 μg/g TAC: 511.18 mg ASE/g extract	Vasyliev et al. (84)
Tomato waste (peels + seeds, dried)	Lycopene, β-carotene, lutein, tocopherol (vitamin E)	Lycopene: 215.13 ± 4.31 μg/g DW β-carotene: 206.95 ± 3.27 μg/g DW Tocopherol: 130.86 ± 8.97 μg/g DW Total lipophilic content: 553.84 ± 16.61 μg/g DW; TEAC: 93.84 ± 0.18 mM Trolox eq	Badea et al. (85)
Orange pomace	Proanthocyanidins, hesperidin, naringin, eriocitrin, luteolin, diosmin, flavonols, hydroxycinnamic acids, limonoids	Proanthocyanidins (DMAC): 0.8 ± 0.2 mg epicatechin/100 g Hesperidin: 229 ± 33 mg/100 g FRAP: 2.6 ± 0.3 mg GSH/g Hydroxyl radical inhibition: 78 ± 8% ABTS scavenging: 54 ± 2%	Plaza and Marina, (86)
Supercritical Fluid Extraction (SFE)			
Tomato pomace	Lycopene ((all-E)- and Z-isomers), tomato seed oil, antioxidant compounds (n.i.)	Extract yield: 142.5–165.3 g/kg DW; lycopene recovery: 85%; lycopene in extract: 1313–1339 μg/g extract; TEAC: 84.3–96.9 μmol Trolox/g extract	Squillace et al. (87)
Jalapeno pepper	Oleoresin, phenolics, flavonoids, fatty acids (linoleic, palmitic, oleic acids), carotenoids (β-carotene, lycopene)	Oleoresin yield: 9.38–10.08% (w/w) TPC: 12.3–16.7 mg GAE/g extract; β-carotene: 0.27–1.26 μg/g extract	Fornereto Soldan et al. (88)
Microwave Assisted Extraction (MAE)			
Raspberry pomace (skin, seeds, pulp)	Gallic acid, protocatechuic acid, 4-hydroxybenzoic acid, p-coumaric acid, vanillic acid, vanillin, ferulic acid, rutin, catechin, epicatechin, quercetin	TPC = 68.10 mg GAE/g DW; TFC = 63.82 mg RE/g d.w. (~2 × conventional); gallic acid (1,955 μg/g, 90% of total phenolics)	Jaouhari et al. (89)
Dried tomato, tomato paste, tomato sauce and dried carrot	Carotenoids (lycopene, β-carotene), phenolic compounds (chlorogenic acid, caffeic acid, rutin, p-coumaric acid, ferulic acid, kaempferol)	Highest TAC: dried tomato= 5.10 mmol lycopene equivalents kg^−1^; partial co-extraction of hydrophilic phenolics with DES; DES recyclable up to 3 times (75–90% recovery)	Görüşük et al. (90)
Apple pomace	Phenolic compounds (n.i.), vitamin C	TPC: 5.21 ± 0.09 mg GAE/g, DPPH: 93 ± 1%; FRAP: 3.22 ± 0.04 mg TEAC/g; Vitamin C: 20.00 ± 0.12 mg/100 g	Bhat et al. (91)
Red grape pomace	Pectin and phenolic extract (gallic acid, chlorogenic acid, caffeic acid, epicatechin, m-coumaric acid, rutin, quercetin, catechin, quercitrin)	GPP yield: 20.25 ± 1.66%; GPE yield: 21.51 ± 1.22%; GalA content: 70.59%; DE: 45.16% (low-methoxyl pectin); dominant phenolic: gallic acid (24.58% of GPE)	Amiri Samani et al. (104)
Pressurized Liquid Extraction (PLE)			
Mango, litchi, jackfruit, pineapple, papaya and banana pomace	Phenolic compounds (n.i.)	TPC: 7.02–237.22 mg GAE/g DW; TFC range: 0.35–21.11 mg QE/g DW; DPPH IC50: 0.16–1.89 mg/ml; ABTS+ IC50: 0.13–1.20 mg/ml; α-glucosidase IC50: 0.15–0.39 mg/ml	Islam et al. (92)
White and red onion scales (inedible) and dried edible interior	Quercetin and other phenolic compounds (n.i.)	Quercetin: 1.81–40.28 mg/g DW; TPC: 19.8–94.6 mg GAE/g DW; quercetin red scales: 5.11–34.0 mg/g DW; TPC red scales: 60.4–119 mg GAE/g DW; DPPH inhibition: up to ~85%; ABTS inhibition: up to ~100%; β-carotene bleaching inhibition: up to ~100%	Olszowy-Tomczyk et al. (93)
Pulse Electric Field Extraction (PEFE)			
Tomato waste (peels + seeds)	Lycopene and others carotenoids (n.i.) Phenolic compounds (n.i.)	Total carotenoids: 15.31–17.77 mg/100 g tomato waste; lycopene: 11.07–14.31 mg/100 g; TPC: 35.33–56.16 mg gallic acid/kg; proteins: 107.4–145.1 mg BCA/100 g; AC: 0.40–0.78 mM Trolox eq./100 g	Andreou et al. (94)
Fresh white grape pomace (skins + seeds)	Epicatechin, p-coumaric acid, and other phenolic compounds (n.i.)	Epicatechin: 1.79 mg/g DW; p-coumaric acid: 0.075 mg/g DW TPC: 4.38 ± 0.30 mg GAE/g DW; FC: 8.16 ± 0.30 mg QE/g DW; FRAP: 3.37 ± 0.07 mg AAE/g DW; epicatechin: 1.79 ± 0.2 mg/g DW; p-coumaric acid: 0.075 ± 0.01 mg/g DW; quercetin: 0.064 ± 0.001 mg/g DW	Carpentieri et al. (95)
Apple pomace	Phloridzin and other phenolic compounds (n.i.)	TPC: 181.4–223.5 μg GAE/g fresh pomace; phloridzin: 753.84 ± 26.38 μg/g	Pollini et al. (96).
Tangerine peel	Hesperidin	Hesperidin: 669.38 μg/ml	Xu et al. (97)
Enzyme-Assisted Extraction (EAE)			
Bilberry pomace	Polyphenols, anthocyanins, soluble saccharides	Water-soluble yield: 56.15 g/100 g DW (~30% increase vs. SLE); TPC: 6.8 mg GAE/g DW; anthocyanins: 1.79 mg cyanidin-3-O-glucoside eq/g DW	Syrpas et al. (98)
Sweet cherry pomace	Non-extractable polyphenols (NEPs); proanthocyanidins	TPC: 1.1–1.7 mg GAE/g sample; PA content (butanol/HCl): 29–43 mg epicatechin/100 g sample	Domínguez-Rodríguez et al. (99)
Blackcurrant waste (lyophilized powder)	Anthocyanins (delphinidin, cyanidin, petunidin, pelargonidin, peonidin glycosides)	Total: 21.99 ± 1.20 mg anthocyanin/g sample	Aliaño-González et al. (100)
High Pressure Processing-Assisted Extraction (HHPE)			
Black carrot pomace	Anthocyanins, total phenolic compounds, antioxidant compounds (n.i.)	TMA: ~105.7 mg cyanidin-3-glucoside eq/l; TPC: ~333.9 mg GAE/l; AA: ~89.3% DPPH inhibition	Agcam et al. (101)
Red wine grape pomace	Phenolic compounds (n.i.)	TPC= 906.34 ± 12.33 mg GAE.100 g^−1^	Cascaes Teles et al. (105)

AAE, ascorbic acid equivalents; ABTS, 2,2′-azino-bis(3-ethylbenzothiazoline-6-sulfonic acid) assay; AC, antioxidant capacity; BCA, bicinchoninic acid protein assay; DE, degree of esterification; DES, deep eutectic solvent; DMAC, 4-(Dimethylamino)cinnamaldehyde assay (proanthocyanidins); DPPH, 2,2-diphenyl-1-picrylhydrazyl assay; DW, dry weight; EAE, enzyme-assisted extraction; FC, flavonoid content; FRAP, ferric reducing antioxidant power assay; GAE, gallic acid equivalents; GalA, galacturonic acid; GPE, grape pomace extract; GPP, grape pomace pectin; GSH, glutathione equivalents; IC50, half maximal inhibitory concentration; n.i., not individually identified; NEPs, non-extractable polyphenols; PA, proanthocyanidins; QE, quercetin equivalents; RE, rutin equivalents; TAC, total antioxidant capacity; TEAC, trolox equivalent antioxidant capacity; TFC, total flavonoid content; TMA, total monomeric anthocyanins; TPC, total phenolic content.

On the other hand, the use of natural deep eutectic solvents (NADESs) instead of toxic and environmentally hazardous solvents like methanol and ethanol is gaining attention (80, 81). NADESs are biodegradable and less harmful, making them an eco-friendly option. They consist of a mixture of hydrogen bond acceptors and donors, whose polarity, diffusivity, viscosity, and conductivity facilitate the extraction process (82). Examples regarding UAE combined with NADES can be seen in Table 1. This strategy consistently outperforms conventional organic solvents in bioactive compound recovery from agri-food byproducts. Choline chloride (ChCl):lactic acid-based NADES improved quercetin and chlorogenic acid yields by up to 92% over conventional ethanol in apple pomace (83). Improved recovery was also reported for phenolic acids in tomato pomace (84), carotenoids and tocopherol in tomato waste (85), and flavonoids in orange pomace (86).

SFE with CO_2_ enables sequential fractionation, for which carotenoids and oils can be selectively recovered first (lycopene recovery up to 85 and oleoresin 10%), leaving phenolic compounds intact for a subsequent extraction step., maximizing total bioactive recovery from a single stream (87, 88).

When MAE is used, phenolic yields can be doubled over conventional methods as demonstrated by Jaouhari et al. (89) in raspberry pomace. It allows also simultaneous lipophilic-hydrophilic fractionation in tomato when combined with NADES (90) and outperforming conventional oven drying in antioxidant preservation in apple pomace (91).

Applying PLE, and specifically its aqueous modality (pressurized hot-water extraction, PHWE), applied to mango peel yielded a total phenolic content (TPC) of 180.12 ± 7.33 mg GAE/g DM, approximately five times higher than conventional organic solvent extraction (92). In onion scales, PHWE similarly outperformed Soxhlet extraction, which reduced quercetin yield due to prolonged thermal exposure, confirming the advantage of controlled pressurized conditions for preserving thermolabile compounds (93).

PEFE can be applied both as pre-treatment prior to solvent extraction and as a direct extraction technique. In the former mode, it increased lycopene by 45% and phenolics by 101% in tomato waste (94), and flavonoid content by 43% in white grape pomace (95). Applied directly, it recovered ca. 754 μg phloridzin/g from apple pomace (96) and improved hesperidin yield (+37%) in tangerine peel over conventional alkali dissolution, without altering the molecular structure of the extract (97).

EAE compared to conventional extraction, can increase water-soluble fraction yield by ca. 30% in bilberry pomace (98), achieved higher proanthocyanidin recovery and antioxidant capacity that acid and alkaline hydrolysis in cherry pomace (99), and yielded equivalent anthocyanins content to UAE (21.64 mg/g) in blackcurrant with shorter extraction times (100).

HHPE using water as solvent achieved total monomeric anthocyanins (TMA) and TPC yields of 105.7 mg/l and 333.9 mg/l from black carrot pomace at optimized conditions, demonstrating that pressure can substitute organic solvent for the extraction of anthocyanins and phenolics (101).

To further improve the efficiency of extraction, some authors advocate a combination of green and emergent technologies (102, 103). Indeed, combined UAE-MAE on red grape pomace enabled simultaneous recovery of a pectin fraction (20, 25%, GalA content 71%) (104). Likewise, when HHP was applied in combination with EAE from red wine grape pomace, it improved the extraction efficiency up to 16-fold, yielding a maximum TPC of 906.34 mg GAE/100 g (105). Similarly, the combined application of HHP and ohmic heating increased total phenolic extraction yield by 98–103% and antioxidant capacity by 35–65% over conventional Soxhlet extraction in prickly pear peel (103).

Despite promising results, these approaches require further research to optimize product yields, lower operational costs, and resolve scale-up challenges. Nonetheless, these technologies represent key tools in developing sustainable, industrial-scale extraction processes aligned with green chemistry and circular economy goals (106–108).

Although the extraction methods summarized in Table 1 were largely validated on processing by-products, their principles are directly transferable to surplus and ugly F&Vs. Critically, green and mild conditions, low temperatures, food-grade solvents, short extraction times, are not only more environmentally sustainable but also more effective at preserving thermolabile bioactive compounds. Food-grade processing should therefore not be regarded as a downgrade from fresh consumption, but as a complementary strategy that retains nutritional value when redistribution is not possible, transforming discarded produce into high-value functional ingredients.

### 3.4 Use for animal feed

Animal feed represents an indirect but still nutritionally meaningful valorization pathway for surplus and ugly F&Vs. Although nutrients are no longer consumed directly by humans, they enter the animals' metabolism and may contribute to nutrient-rich food for human consumption (109, 110).

The conversion of FL into animal feed can range from straightforward processes, such as directly feeding fresh culled F&Vs or grinding and ensiling with fibrous substrate (grass, straw, hay, or crop residues), to more complex treatments including chemical and physical processing, insect bioconversion, fungal bioprocessing, microbial fermentation and even anaerobic digestion (58, 110, 111).

Baker et al. (112) demonstrated that FL from citrus was employed to feed dairy cattle instead of conventional ingredients. This action reduced costs, fertilizers and pesticides use, GHG emissions, water consumption, and decreased the use of arable land to feed animals, all while keeping stable animal production levels and promoting the circular economy between agriculture and livestock farming. However, large quantities of FL can pose challenges, such as accumulation and deterioration, since simultaneous feeding to animals is often impractical. The option to ensile FL from F&Vs with dry substrate allows for storage of up to 200 days while maintaining the feeding value according to Monllor et al. (113), or even 3 years as reported by certain enterprises in the sector. This ensiling technique consists of fermenting F&Vs with base dry substrate under anaerobic conditions to produce a stable, nutrient-rich feed. Overall, the economic viability can be further impacted by the minimal sell price of these F&Vs or the lack of economic feasibility studies, making other options within the FWH, such as material recycling, more attractive to farmers (19).

While animal feed occupies an intermediate position within the FUWH, its nutritional relevance distinguishes it from material recycling or energy recovery, justifying its consideration as a preferable option when human food applications are not viable. However, nutritional efficiency losses are inherent to trophic conversion, and feed use should therefore be prioritized only after food-grade options have been exhausted.

### 3.5 Recycling and nutrient recovery

Moving further down the hierarchy, this tier encompasses processes aimed at recovering nutrients and organic matter from FL which can no longer enter the food chain. These include soil amendment through tillage incorporation (till back), biological decomposition via composting and vermicomposting, extraction of high-valuable compounds, and nutrient recovery through anaerobic digestion to produce biogas and digestate as biofertilizers.

#### 3.5.1 Till back

F&Vs that are not collected (i.e., non-harvested or rejected on-site) may be tilled back into the soil, reintegrating organic matter into the natural system and contributing to nutrient cycling.

Tilling abandoned F&Vs into the soil is often seen as one of the easiest and most cost-effective methods for their disposal. Farmers often see this practice as positive or, at minimum, with a neutral environmental impact. The incorporation of freshly chopped plant material into the soil enhances its physical structure, chemical composition, and biological activity. Additionally, it provides a fertilizing effect, enabling a significant reduction in the need for supplemental fertilizers during subsequent cultivation (114). Other plant residues, such as leaves and branches, can also be incorporated alongside F&Vs, provided they are fresh.

Although fresh F&Vs can be tilled into the soil, this must occur within 24 h to prevent degradation issues. In some cases, biosolarization may be necessary following incorporation to control a wide range of soil pathogens, enhance crop health, and improve soil fertility by incorporating organic amendments (like crop residues, manure or compost) (115).

#### 3.5.2 Composting and vermicomposting

Composting is an aerobic, microbially driven process that transforms organic waste into a stable, humified product. In contrast, vermicomposting utilizes earthworms (such as *Eisenia fetida*) in conjunction with microorganisms, resulting in a product with higher levels of nitrogen, phosphorus, potassium, and micronutrients (116). Unlike traditional composting, vermicomposting does not involve a thermophilic stage but instead operates at moderate temperatures suitable for earthworm activity, which makes it more sensitive to environmental factors such as temperature, humidity and oxygen (117). While both processes effectively reduce the organic waste load destined for landfills and offer positive environmental impacts, vermicomposting is generally more effective on a small scale and is considered more environmentally friendly due to lower greenhouse gas (GHG) emissions (118). Nevertheless, proper management of both methods is essential to minimize emissions and prevent water pollution caused by eutrophication, a process where excessive nutrients in water bodies lead to overgrowth of algae, which can harm aquatic life and degrade water quality (119). Ultimately, composting is often more suitable for large-scale, cost-effective applications, whereas vermicomposting is better suited for small- to medium-scale operations, allowing farmers to select the method that best fits their specific needs and resources.

#### 3.5.3 Non-food biorefineries

In this tier, it should be emphasized that, unlike Section 3.3., which addresses the extraction of food-grade bioactive and nutritional compounds for human consumption, the biorefinery model discussed here operates at a lower tier of the FUWH, valorizing produce that can no longer enter the food chain into non-food outputs. This approach represents a transformative strategy for abandoned F&Vs in the primary agricultural sector, converting residues into high-value products such as bio-based chemicals, bioplastics, colorants, enzymes, proteins, cosmetics and pharmaceuticals (120). This conceptual distinction is essential to avoid conflating two strategies that differ fundamentally in their nutritional ambition and hierarchical priority.

#### 3.5.4 Nutrient recovery through anaerobic digestion

Anaerobic digestion utilizes microbial activity to manage organic waste, producing biogas while generating nutrient-rich digestate (121). Only the digestate falls within the nutrient recovery category, while biogas is considered energy recovery.

Unlike lignocellulosic biomass (straw, wood, agricultural waste, municipal solid waste), F&Vs are a renewable energy resource with a different chemical composition. Their higher pectin and lower lignin content eliminate the need for severe pretreatment (121). Pectin, being water soluble, is easily degraded into monosaccharides for ethanol production. However, the fermentation of pectin is more difficult than that of cellulose or hemicellulose. Because of these differences, F&Vs may require adapted pretreatment and enzymatic hydrolysis methods to optimize their use in the production of bioenergy and, consequently, biofertilizer (122).

### 3.6 Energy recovery

Biofuels, classified as liquid (e.g., bioethanol) or gaseous (e.g., biomethane), are produced through fermentation and anaerobic digestion. Pectin-rich agri-FL are valuable for biofuel production due to their fermentable sugars and low lignin content. However, pectin's chemical properties make it resistant to fermentation by most yeasts, including *Saccharomyces cerevisiae*. Pre-extracting valuable compounds like phytochemicals and pectin can also facilitate biofuel production through simpler hydrothermal and enzymatic processes (123). The biorefinery process for these wastes typically involves extracting essential oils and pectin, followed by enzymatic hydrolysis and fermentation or anaerobic digestion to produce biofuels (124).

Although this tier aimed at energy recovery, as well as the previous one at material recycling may offer environmental benefits, such as returning nutrients to the soil or generating renewable energy, they result in the irreversible loss of food-grade nutritional value for human consumption. From a nutrition-sensitive perspective, reliance on lower-tier recovery options for edible F&Vs reflects a suboptimal use of resources. Considering nutritional trade-offs in valorization decisions highlights the need for timely and appropriate actions to prevent unnecessary losses of edible food.

### 3.7 Disposal

The disposal of agricultural and agro-industrial waste remains a critical environmental challenge, mainly due to inadequate management practices such as open dumping, burning, or uncontrolled landfilling. These methods are still widely reported as the most common fates for untreated and underutilized agricultural residues (125, 126). Such practices not only represent a significant loss of potentially valuable biomass but also contribute to the GHG emissions, thereby intensifying climate change. Furthermore, the degradation of organic matter under uncontrolled conditions can lead to the formation and release of other harmful gaseous byproducts, including volatile organic compounds (VOCs) and ammonia, which may contribute to air pollution and pose risks to both human health and ecosystem stability. In addition to atmospheric impacts, the careless disposal of agro-waste can result in soil and water contamination through nutrient leaching and accumulation of hazardous compounds. These cumulative effects highlight the urgency of implementing sustainable and circular approaches to agricultural waste management.

Finally, from a nutrition-sensitive perspective, disposal represents the irreversible elimination of any potential nutritional contribution to food systems and should only be considered when all other options have been exhausted for safety or logistical reasons.

## 4 Challenges and future perspectives for FUWH implementation

### 4.1 Regulatory, logistical and economic barriers

Despite the availability of valorization strategies at every tier of the FUWH, their adoption at the farm level is limited. A recurring theme in the literature is the significant gap between the theoretical potential of these strategies and their practical implementation, which is driven by a combination of regulatory, logistical, and economic barriers. Understanding these barriers is essential for designing effective policy interventions that align farmer incentives with nutrition-sensitive food system goals.

**Figure 3:**
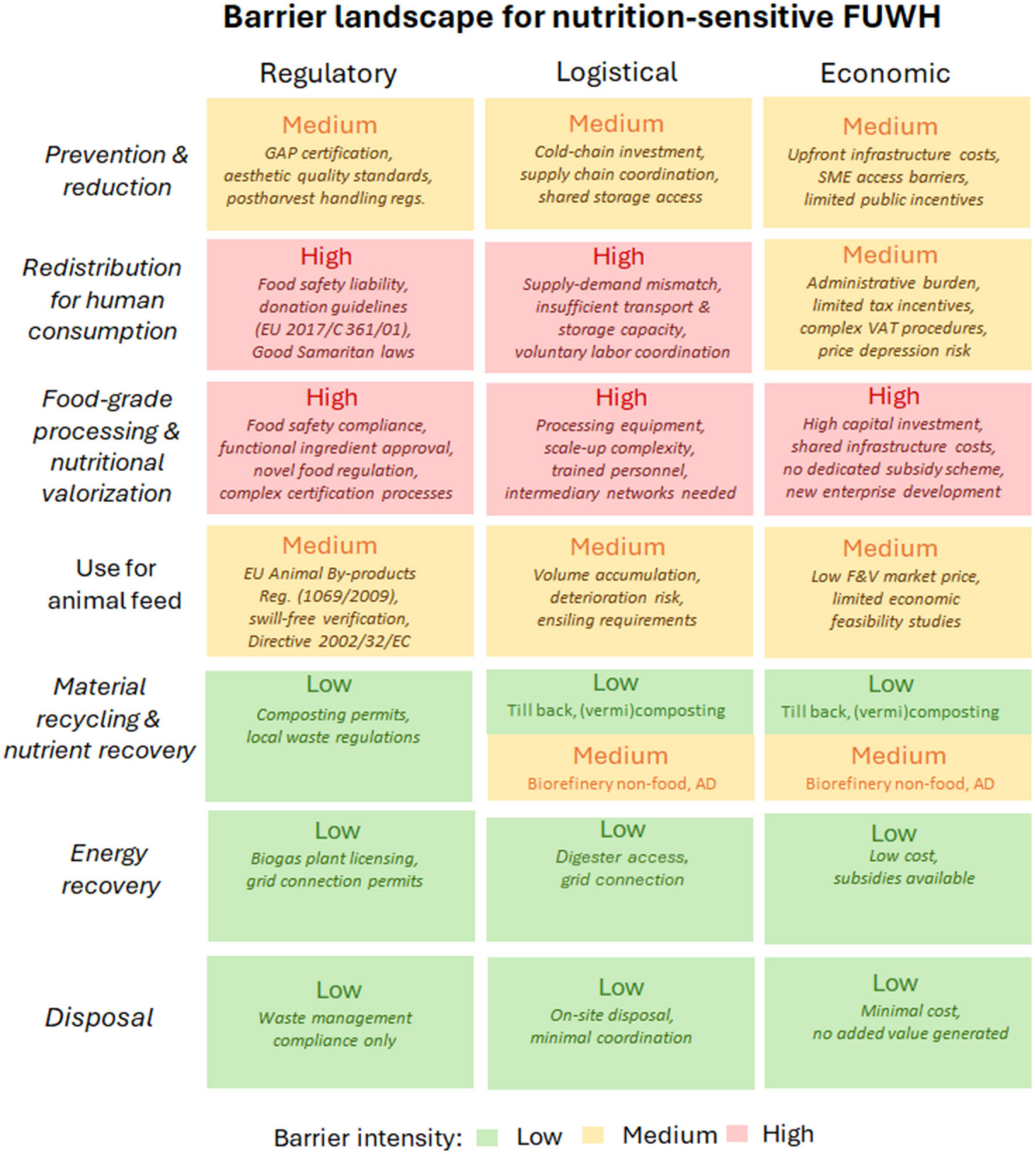
Barrier landscape for nutrition-sensitive valorization across the EU Food Use and Waste Hierarchy. Intensity of regulatory, logistical, and economic barriers at each Food Waste Hierarchy tier, based on a qualitative synthesis of the literature reviewed in Section 3.

As illustrated in Figure 3, the intensity of regulatory, logistical, and economic barriers varies markedly across the tiers of the FUWH, revealing a nutrition-sensitive paradox. Strategies with the highest potential to preserve nutritional value, redistribution for human consumption and food-grade processing, face the highest implementation barriers. Conversely, lower-tier options are more accessible to farmers. This inverse relationship between nutritional value and implementation feasibility highlights the necessity of targeted policy interventions that reduce barriers at the upper tiers of the hierarchy instead of resorting to lower-tier alternatives.

#### 4.1.1 Regulatory barriers

Health standards and product liability represent one of the most frequently cited deterrents to participation in FRAs. Farmers express concern regarding the pursuit of food safety certifications, such as Good Agricultural Practices certification (in the US) or EU Regulation (EC) No 852/2004 (127) on the Hygiene of Foodstuffs (in Europe). Liability issues are also prominent, as the stringent EU food safety legislation and food donation guidelines EU 2017/C 361/01 (128) present significant compliance challenges for growers (4). In response to this trend, EU countries such as Italy, since 2003, have supported and legalized this practice under the *Good Samaritan law*, whereby donors commit to providing safe produce but are absolved of responsibility in cases where recipients mismanage donations (58, 67).

For animal feed use, compliance with EU Animal By-products Regulations (Regulation 1774/2002 and 1069/2009) and Directive 2002/32/EC (129, 130) adds regulatory complexity, although these frameworks generally permit the use of farm-level FL as animal feed, provided that the raw materials are verified to be free from swill, specifically excluding meat, fish, or any other animal-derived products.

Food-grade processing and nutritional valorization face additional regulatory complexity, including compliance with food safety legislation (127), traceability and labeling requirements, and, where novel extraction processes or concentrated bioactive fractions are involved, potential pre-market authorization under Regulation (EU) 2015/2283 on novel foods (131).

#### 4.1.2 Logistical barriers

Logistical limitations are a major constraint across multiple FUWH tiers. Innovative distribution models, such as HFHs and collaborative logistics networks, have emerged as solutions to reduce logistical costs and improve supply chain efficiency. However, their implementation requires coordination between multiple actors and is not yet available in many production regions.

As discussed in Section 3.2, at the redistribution level, the supply-demand imbalance between agricultural donations and food bank needs results in periods of oversupply or shortages, compounded by insufficient storage and transport capacity and the challenge of coordinating voluntary labor,.

For use as animal feed, large volumes of FL can pose challenges related to accumulation and deterioration since simultaneous feeding is often impractical. Therefore, it is necessary to consider ensiling FL from F&Vs with dry substrate that allows for storage of up to 200 days while maintaining feeding value (113), making it a practical solution for managing large volumes.

For food-grade processing, key logistical barriers include the lack of accessible processing equipment at farm level, the complexity of scale-up, and the absence of intermediary networks connecting the edible discarded produce with transformation facilities.

#### 4.1.3 Economic barriers

Economic viability is a central concern for farmers considering any strategy beyond routine disposal. Bureaucratic challenges primarily revolve around the complexity of administrative processes associated with accessing economic incentives, value added taxes (VAT) exemptions, tax credits, and market-management schemes under Commission Delegated Regulation (EU) 2017/891 (132), which many SMEs find too burdensome to manage and abandon the produce beforehand in the field (14). Even farmers who successfully accessed incentives, such as tax credits, reported that these measures had limited impact on their decision to donate, as production costs far exceeded the compensation received (133, 134).

Additionally, farmers actively participating in FRAs expressed concern that donating surplus could drive down crop prices at the farm stage, potentially harming the broader industry. For material recycling and biorefinery options, the minimal sell price of F&Vs or the lack of economic feasibility studies makes lower-tier options more attractive to many farmers (19, 135).

To address these economic barriers, a combination of simplified administrative processes, transparent and effective financial incentives, and the distribution of the financial burden of adopting new practices among beneficiaries, including governments, organizations, and other stakeholders, via subsidies, grants, or co-financing programs is required. However, overcoming the aforementioned barriers remains a complex challenge, as economic, institutional, and behavioral factors are often deeply interconnected. Therefore, further research is needed to evaluate the effectiveness of different policy instruments, identify context-specific solutions, and develop scalable frameworks that can be adapted across regions and sectors.

Reaching food-grade transformation requires not only technological investment but also trained personnel, shared infrastructure, and new intermediary enterprises bridging farmers and functional ingredient markets. Targeted policy support is therefore essential, including co-financed investment programs and technical advisory services integrated into existing agricultural extension networks.

### 4.2 Key considerations and future directions

Despite the increasing availability of information, there is a lack of clarity regarding the full range of strategies that farmers can implement to reduce FL while adhering to the EU FUWH. Furthermore, studies based on interviews reveal discrepancies between policymakers and farmers, accompanied by widespread uncertainty about the effectiveness of current policies (7, 14, 133). Indeed, the priorities of governments or policymakers do not always reflect the perspectives and needs of farmers. Fostering trust and reliability is key to encouraging farmers to comply more willingly with these strategies.

The most frequently cited concerns were external factors affecting the marketability of produce, including market fluctuations that generate surpluses and rejection for ugly produce. To address these challenges, stakeholders in the early stages of the FSC would benefit from initiatives aimed at stabilizing market conditions and prices, as well as increasing consumer acceptance and demand for imperfect products. In conjunction with food business operators, governments and public authorities should encourage the development of new market opportunities for ugly products and advocate for the relaxation of aesthetic quality standards.

The lack of logistical and economic feasibility assessments when adopting these strategies is also a recurring concern, particularly in SMEs. Many of the presented strategies return economic benefits in the medium to long term through the reduction of FL, increased productivity, and the reduction of disposal costs. Nonetheless, implementing them often incurs significant upfront operational costs that must be carefully assessed, as well as potential reliance on government incentives to offset the initial investment. While some lower-tier strategies may appear more affordable or offer greater added value in the short term, it is essential to weigh environmental and social sustainability along with ethical implications, especially when higher-tier options like prevention are available.

It is also important to note that, for a real and just agrifood transition toward near-zero FLW, this compromise must be proportionate to the capacities of different stakeholders. SMEs should not be expected to bear the same level of responsibility as larger enterprises. Other groups can also be considered vulnerable and may require tailored attention, including farmers in low-income countries and regions, elderly farmers lacking prospects for farm succession, and small-holder farmers particularly exposed to technological shifts. Policy and support are therefore critical in helping producers implement FUWH strategies.

## 5 Conclusions

This review presents a nutrition-sensitive adaptation of the EU FUWH, extending its scope beyond environmental and economic criteria to incorporate nutritional value, a dimension absent from the original framework. Embedding nutritional criteria into valorization decision-making represents a practical step toward aligning food system policies with both sustainability and public health objectives.

While these strategies offer promising solutions, they also involve significant trade-offs and implementation challenges. Their effectiveness depends on farm-level actions as well as broader market dynamics and institutional conditions. Therefore, policy support is essential to enable their adoption. This support should include fair and stable pricing mechanisms that cover production costs and allow farmers to invest in alternative strategies. This must be complemented by structural measures such as investing in shared logistics and cold-chain infrastructure, providing technology and technical advisory services to smallholders, and creating platforms that connect discarded produce with redistribution networks and food-grade processors. Reducing aesthetic quality standards is also necessary to facilitate the valorization of ugly produce.

Access to clear and practical information remains a major constraint for many primary producers, whose willingness to adopt new strategies is often limited by uncertainty rather than lack of interest. Ensuring that the transition toward more sustainable food systems is both effective and equitable also requires identifying and addressing the specific vulnerabilities of the most exposed producers.
